# TCMRGAT: Relational graph attention networks for predicting stroke treatment efficacy of traditional Chinese medicine prescriptions

**DOI:** 10.3389/fphar.2025.1570094

**Published:** 2025-12-02

**Authors:** Ning Cheng, Changsong Ding, Xuekun Song

**Affiliations:** 1 School of Information Technology, Henan University of Chinese Medicine, Zhengzhou, China; 2 School of Informatics, Hunan University of Chinese Medicine, Changsha, China; 3 Big Data Analysis Laboratory of Traditional Chinese Medicine, Hunan University of Chinese Medicine, Changsha, China

**Keywords:** traditional Chinese Medicine, stroke treatment, prescriptions prediction, relational graph attention network, generative adversarial network, prescriptions efficacy

## Abstract

**Background:**

Stroke is a serious neurological disorder that poses a global health challenge. Traditional Chinese Medicine (TCM) prescriptions have shown potential in its treatment. However, TCM prescriptions typically involve a wide variety of botanical drugs, and the efficacy of different combinations varies, with underlying patterns remaining unclear. This study aims to develop a model to predict the efficacy of TCM prescriptions for stroke, so as to deepen understanding of the underlying mechanisms of botanical drug therapies.

**Methods:**

We collected stroke-related TCM data, including prescriptions, botanical drugs, metabolites, and targets, from TCM classics and the HERB database. A generative adversarial network (GAN) was used to augment imbalanced data, and constructed a heterogeneous network. Then, we initialized node features and performed neighborhood feature learning using a relational graph attention network (RGAT) to predict TCM prescription efficacy. We compared our method, named RGAT for TCM prescription efficacy prediction (TCMRGAT), with other models.

**Results:**

TCMRGAT achieved an accuracy of 0.843 and an area under curve (AUC) of 0.853 on balanced data, outperforming competing methods. Ablation experiments confirmed the effectiveness of GAN-based data augmentation. Case studies using RGAT and GPT-4 highlighted the model’s potential in real-world applications. Analysis of post-training attention weight changes revealed potential key botanical drug-metabolite relationships, suggesting they may be directly associated with stroke treatment.

**Conclusion:**

TCMRGAT aids in predicting prescription efficacy and identifying key metabolite s for stroke treatment. This study provides valuable insights into the use of Traditional Chinese Medicine for stroke and offers a promising direction for future research.

## 1 Introduction

Stroke is a serious condition that presents significant risks to human health (Langhorne et al., 2011), with high morbidity, disability, and mortality rates, which impose a heavy strain on individuals, families, and healthcare systems. (Kanazawa et al., 2019; Wafa et al., 2020). Although drugs used in Western medicine are effective in treating stroke, they may lead to side effects and pose various risks, including bleeding (Yedong et al., 2024), liver and kidney damage (Mohammed et al., 2023) and gastrointestinal issues (Guo et al., 2022). Additionally, treatment plans must be individualized, as they are not universally effective (Ton et al., 2019; Wu et al., 2019). Traditional Chinese Medicine has a long history of understanding and treating stroke, dating back to the “Huangdi Neijing”. It emphasizes syndrome differentiation and tailors individualized treatment plans according to patients’ symptoms, constitutions, and disease stages (Ton et al., 2019). Ancient physicians developed several prescriptions for stroke, including Buyang Huanwu Decoction, Zhengan Xifeng Decoction, and Tianma Gouteng Decoction, employing methods such as tonifying qi-flowing, promoting blood circulation, suppressing hyperactive liver for calming endogenous wind, and expelling phlegm (Zhang W. et al, 2024). The use of TCM in stroke treatment is well-established in clinical practice (Ton et al., 2019). Studies have shown that certain TCMs exhibit effects such as anti-platelet aggregation, antioxidation, and nerve cell protection, offering a scientific foundation (Fan et al., 2024). However, these medicines contain numerous pharmacologically active metabolites with unclear mechanisms, and their combined efficacy demonstrates a non-linear relationship with their properties (Cheng et al., 2021). Effective methods for thoroughly analyzing its scientific connotations are lacking, which makes constructing stroke prescriptions based on botanical drug attributes difficult and impedes further research in stroke treatment. As the efficacy of prescriptions is linked to their therapeutic symptoms, predicting the effectiveness of prescriptions composed of different botanical drugs could assist in identifying potential stroke treatments. Therefore, prescription efficacy prediction serves as a valuable tool in advancing the research on the therapeutic effects of TCM prescriptions for stroke.

Prescription efficacy prediction is fundamentally a study of the compatibility principles in TCM. Compatibility, as the foundation of compound prescriptions, is essential for understanding their underlying mechanisms (Wang et al., 2021). It leverages the synergy of pharmacologically active substances to enhance efficacy and minimize toxicity. Recently, with the integration of artificial intelligence and TCM, bioinformatics has been combined with the complex systems of TCM to explore the pharmacodynamic basis and mechanisms at the molecular network level. Computer programming facilitates the prediction and validation of complex mechanisms (Pan et al., 2024). Additionally, the integration of knowledge graphs and deep learning enhances the accuracy of drug predictions (Zhang et al., 2023; Zitnik et al., 2018). Graph neural networks, combining deep learning with graph computing, can uncover underlying patterns between entities by leveraging relationships and processing biological information from prescriptions, thus advancing drug interaction prediction (Ryu et al., 2018; He et al., 2022). Various modern information technologies are increasingly utilized in TCM compatibility research (Li et al., 2022). Current data mining and prediction tasks predominantly rely on network pharmacology analysis, neglecting the integration of TCM properties and pharmacodynamic information. Existing models often focus on integrating multiple data sources or using only TCM features, with limited attention paid to the potential correlations between TCM and other biological entities (Liu et al., 2018). Few studies explore TCM compatibility principles by combining TCM characteristics with biological data.

Constructing heterogeneous biological information networks is an emerging field in bioinformatics, aiming to integrate various biological data sources into a unified network for a more comprehensive understanding of biological system complexity. Some studies have combined data from genomics, proteomics, and metabolomics to uncover molecular interactions and regulatory mechanisms (Yang et al., 2022). Network representation learning techniques can extract entity features from complex networks and represent them as low-dimensional dense vectors, effectively supporting downstream tasks like node classification (Bi et al., 2022) and clustering (Zhao et al., 2019), link prediction (Liao et al., 2022), and graph classification (Ma et al., 2021).

Compared to traditional statistical methods, transforming TCM data into graph representations through deep learning enables a quantitative exploration of the underlying patterns in TCM prescriptions from multiple perspectives. For instance, Huang et al. (Huang et al., 2020) developed a pediatric cough symptom network to examine the relationship between pediatric cough symptoms and their prognosis. Zhou et al. (Zhou et al., 2009) represented prescriptions as nodes and constructed a “prescription-prescription” network based on the similarity of prescriptions, which was determined by factors such as “the number of common metabolites” and “prescription length,” to identify core botanical drug combinations. Recently, Graph Neural Network (GNN), a class of deep learning-based methods for processing graph data, has proven effective in capturing network structure information. Due to their superior performance, GNN has found increasing applications in the field of TCM, including intelligent diagnosis (Ruan et al., 2021), meridian prediction (Yeh et al., 2020), prescription recommendation (Jin et al., 2020; Yang et al., 2024; Dong et al., 2024), and prediction of botanical drug-target (Zhao et al., 2021; Duan et al., 2024; Zhang L. et al, 2024) and botanical drug-disease interactions (Hu et al., 2023; Hu et al., 2024). The practical success of GNNs in analyzing large datasets has been well-documented, offering a novel approach for studying the compatibility laws of TCM and a promising method for predicting prescription efficacy.

In this study, we introduce a prescription efficacy prediction model that employs generative adversarial network (GAN) for data augmentation and relational graph attention network (RGAT) (Busbridge et al., 2019) for neighborhood feature learning in heterogeneous graphs. The model comprises five key metabolites: a feature extraction module, a data augmentation module, a heterogeneous graph construction module, a message passing module, and a prediction module. Firstly, botanical drugs and prescriptions are quantified based on properties, flavors, and meridians, while the simple contrastive sentence embedding (SimCSE) framework is used to extract features from simplified molecular input line entry system (SMILES) sequences and protein sequences. Besides, the molecular extended connectivity fingerprints (ECFP) feature (Morgan, 1965), also known as a Morgan fingerprint, is used as an additional feature. Subsequently, GAN is used for data augmentation to address the sample imbalance issue, enhancing the model’s generalization. Using both the original and GAN-generated data, heterogeneous network encompassing prescriptions, botanical drugs, metabolites, and targets is constructed. Then, RGAT is employed for neighborhood feature learning. Finally, a multilayer perceptron (MLP) (Taud and Mas, 2018) is applied to predict prescription efficacy. Our model effectively captures the relationships between TCM and other biological entities through the heterogeneous network. Additionally, the use of GAN for data generation has been shown to improve model performance. Moreover, attention coefficients are incorporated to enhance the model’s interpretability.

Our contributions are outlined below:

1. We collect and sort out relatively comprehensive ancient and modern common prescriptions for stroke treatment, and collected TCM data related to stroke from the HERB database (Gao et al., 2025), including botanical drugs, botanical drug metabolites, and their corresponding targets. A heterogeneous network is constructed, which includes a greater variety of node relationships, and a broader scope compared to existing stroke-related networks (Yang et al., 2021).
2. To address the imbalance in prescription efficacy within the heterogeneous network, we propose a data augmentation method utilizing GAN. This approach demonstrates superior generalization compared to traditional augmentation techniques.
3. We introduce TCMRGAT, a novel model for prescription efficacy prediction, which learns node neighborhood features within a heterogeneous network. Additionally, we analyze the post-training attention weights, which improve the model’s interpretability.
4. We evaluate our model on both balanced and unbalanced datasets. The results indicate that our model outperforms several existing approaches in predicting prescription efficacy. Furthermore, case studies confirm its reliability in real-world prescription efficacy prediction.

## 2 Materials and methods

### 2.1 Data source

This paper has a comprehensive collection of prescriptions for stroke treatment, spanning both ancient and modern approaches. It covers conditions such as transient ischemic attack, cerebral hemorrhage, cerebral infarction, stroke complications, and sequelae. The prescriptions are drawn from classical Chinese medical texts, including the “Treatise on Febrile Diseases”, “Synopsis of Prescriptions of the Golden Chamber”, “Treatise on Differentiation and Treatment of Epidemic Febrile Diseases”, and “Supplement to Thousand Golden Prescriptions”, as well as contemporary Chinese medicine journals like the “Journal of Traditional Chinese Medicine”, “China Journal of Traditional Chinese Medicine”, and “China Journal of Chinese Materia Medica”, published from the 1980s to the present. The data includes prescription names, constituent botanical drugs, and their associated therapeutic effects, encompassing a total of 2,301 prescriptions. Additionally, we have gathered information on botanical drug metabolites from the HERB database (Gao et al., 2025), along with their corresponding targets, SMILES sequences, and protein sequences. Established in 2021, the HERB database integrates multiple TCM databases and incorporates data from published studies that are not yet available in existing TCM resources. It offers a comprehensive collection of botanical drugs and their metabolites. By leveraging data mining and statistical methods, the HERB database has mapped relationships between 12,933 targets, 28,212 diseases, 7,263 Traditional Chinese Medicine, and 49,258 metabolites, offering strong data support for theoretical TCM research.

### 2.2 Heterogeneous graph related data

#### 2.2.1 Prescription-botanical drug

A prescription typically consists of various botanical drugs. The relationship between prescriptions and botanical drugs is derived from our collection of 2,301 prescriptions for stroke treatment in ancient and modern times. Through the botanical drugs contained in the prescription, the relationship between the prescription and botanical drugs is constructed. In total, 26,453 prescription-botanical drug relationships were identified, consisting of 2,301 stroke-related prescriptions and 710 distinct botanical drugs. Each prescription is denoted by its name, while the botanical drugs are represented by their common medicine names.

#### 2.2.2 Botanical drug-metabolite

Botanical drug plays a crucial role in TCM prescriptions. Various combinations of botanical drugs yield distinct therapeutic effects. The attribute data and relationships with metabolites are primarily derived from the HERB database. We gathered targets of stroke-related botanical drugs, resulting in 58,128 botanical drug-metabolite relationships, which encompass 19,709 metabolites, each identified by a unique ID.

#### 2.2.3 Metabolite-target

Different metabolites interact with distinct targets, and metabolites targeting different entities typically exhibit varying therapeutic effects. The metabolite-target relationship is primarily derived from the HERB database. By gathering the targets associated with these metabolites, we identified 176,120 metabolite-target relationships, encompassing 12,933 targets, each identified by a unique ID.

### 2.3 Data cleaning

A total of 2,301 prescriptions is collected, representing 19 distinct efficacies, specifically including invigoration, regulating blood, dispelling wind, expelling phlegm, carbuncle, eliminating dampness, regulating qi-flowing, warming interior, reconciliation, clearing heat, emergency, tranquillization, improving eyesight, purgation, resuscitating, resolving food, astringing, relieve exterior syndrome, moistening dryness. However, 12 of these efficacies account for less than 15% of the total samples. To improve the accuracy and reliability of the prediction results and mitigate the effects of data imbalance, we exclude the underrepresented prescription samples. After this adjustment, there are seven efficacies remain. The sample distribution is provided in Table 1.

**TABLE 1:** Prescriptions data after cleaning.

Prescription efficacy	Positive	Negative
Regulating blood	1,171	1,130
Expelling phlegm	780	1,521
Invigoration	773	1,528
Regulating qi-flowing	765	1,536
Dispelling wind	489	1812
Clearing heat	487	1814
Eliminating dampness	347	1954

The features of metabolites and targets are extracted from the SMILES representations of metabolites and the protein sequences of targets. However, some metabolites and targets lack corresponding sequences in the database. As a result, these nodes and their relationships are removed from the heterogeneous graph. The cleaned data is presented in Table 2.

**TABLE 2:** Heterogeneous graph data before and after cleaning.

Name	Before cleaning	After cleaning
Prescription nodes	2,301	2,301
Botanical drug nodes	710	710
Metabolite nodes	19,709	16,817
Target nodes	12,933	10,112
Prescription-botanical drug relation	23,118	23,118
Botanical drug-metabolite relation	58,128	53,755
Metabolite-target relation	176,120	109,896

### 2.4 Data augmentation

As shown in Table 1, after removing certain prescription efficacies, an imbalance between positive and negative samples persists for some prescription efficacies. Additionally, given the relatively small sample size, both the quantity and distribution of samples are unfavorable for model training (Apu et al., 2025). As a result, the model may suffer from overfitting and exhibit bias in its predictions. To address these issues, some form of data augmentation is necessary. Since the features of the predicted prescriptions are learned through training on a heterogeneous graph, traditional data augmentation methods, such as the synthetic minority oversampling technique (SMOTE), can enhance the prescription data but cannot integrate the augmented data into the heterogeneous graph. Therefore, we propose a heterogeneous graph data augmentation method based on GAN. Through GAN training, we obtain enhanced prescription efficacy, prescription-botanical drug relationships, and prescription features. The number of positive and negative samples after prescription efficacy augmentation and prescription-botanical drug relationship augmentation is shown in Table 3. For prescription efficacies with severe imbalances, such as dispelling wind, clearing heat, and eliminating dampness, we generate 4,000 prescription data. For those with mild imbalances, we generate 2,000 prescription data.

**TABLE 3:** Enhanced prescription data.

Prescription efficacy	Positive	Negative	Prescription-botanical drug relation
Expelling phlegm	1810	2,491	66,647
Invigoration	1931	2,370	95,975
Regulating qi-flowing	1973	2,328	71,434
Dispelling wind	2,686	3,615	92,599
Clearing heat	3,041	3,260	104,125
Eliminating dampness	2,419	3,882	72,245

### 2.5 Heterogeneous graph construction

After data cleaning, we identify four types of nodes and three types of relationships. Each node has corresponding initial features. The initial feature of the botanical drug node is obtained by encoding its properties, flavors, and meridian tropisms using one-hot encoding. The initial feature of the prescription node is derived by summing the initial features of the botanical drugs contained in the prescription. The initial feature of the target is extracted from the protein sequence using the SimCSE model. For the features of metabolites, we extract them using both the SimCSE model and Morgan fingerprints. Morgan fingerprint features are a widely used approach for molecular characterization (Morgan, 1965). We then introduce a virtual node representing the Morgan features of metabolites and connect it to the metabolite node. Feature fusion of the metabolite node is achieved through information transmission within the heterogeneous graph. Subsequently, based on the relationships among the three types of nodes (prescription-botanical drug, botanical drug-metabolite, metabolite-target), we construct a heterogeneous graph. Notably, none of the edges have associated attributes.

### 2.6 Framework designs

**FIGURE 1:**
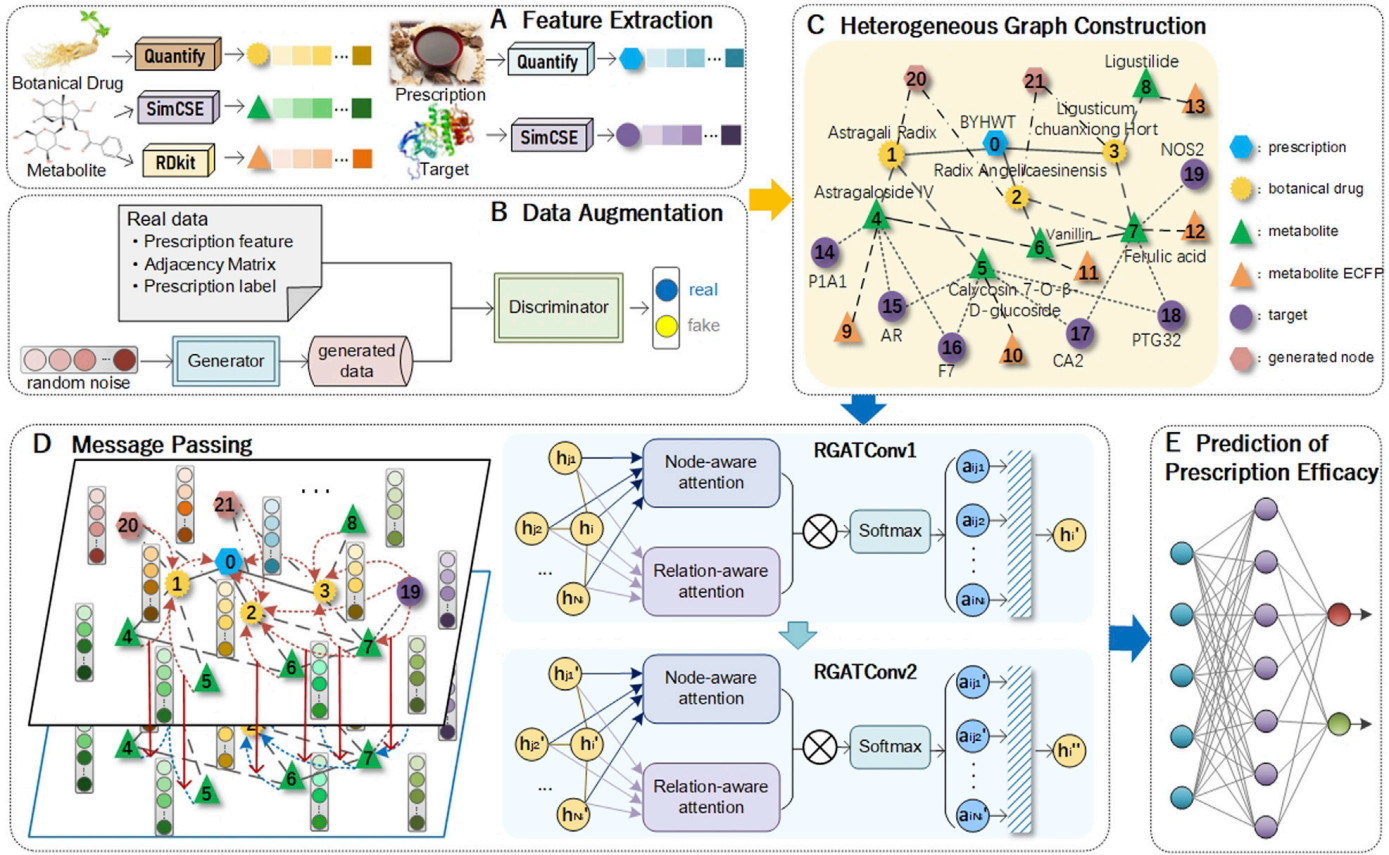
Architecture of TCMRGAT. **(A)** Features initialization extraction of different nodes. The features of botanical drugs are quantified based on their properties, flavors, and meridian tropisms. Prescription features are derived by aggregating the features of their constituent botanical drugs. Metabolite features are extracted from SMILES sequences using SimSCE and RDKit. Target features are obtained from protein sequences through the SimCSE. **(B)** To address data imbalance, GAN is utilized to generate synthetic prescription features, adjacency matrices, and efficacy attributes, providing a balanced foundation for subsequent analyses. **(C)** Heterogeneous graph. The heterogeneous graph comprises six types of nodes: prescriptions, botanical drugs, metabolites, targets, prescription nodes generated through data augmentation, and ECFP feature nodes of introduced metabolites. **(D)** Message passing on the heterogeneous graph. All nodes in the heterogeneous graph have initial features, and then features are extracted through two layers of RGAT. **(E)** Prediction of prescription efficacy. The prescription representations derived from the trained RGAT are utilized as input to MLP to predict prescription efficacy.

This section introduces a model for predicting prescription efficacy. The model framework is illustrated in Figure 1, and it is divided into the following steps.

#### 2.6.1 Feature extraction

Property, flavor, and meridian tropism are essential metabolites of TCM theory. Property and flavor reflect the therapeutic tendencies of botanical drugs. For example, medicinal properties such as cold, hot, warm, cool, and neutral, along with flavors like pungent, sweet, sour, bitter, and salty, each serve specific therapeutic functions. Meridian tropism refers to the selective effects of botanical drugs on specific body parts. In clinical practice, properties, flavors, and meridian tropisms are vital for guiding syndrome differentiation and treatment. They enable medicine to target the affected area precisely, thereby enhancing efficacy and minimizing adverse reactions. In botanical drug medicine combinations, these elements contribute to improved efficacy. During efficacy evaluation, they assist in assessing treatment outcomes and adjusting treatment plans. Therefore, properties, flavors, and meridian tropisms are crucial for understanding and applying Traditional Chinese Medicines, and they also reflect the characteristics of the botanical drugs. Thus, the properties, flavors, and meridian tropisms are selected as the characteristics of botanical drugs. We have identified ten properties and flavors: cold, hot, warm, cool, neutral, sour, bitter, sweet, pungent, and salty. In addition, twelve meridian tropisms are considered: lung, pericardium, heart, large intestine, triple energizer, small intestine, stomach, gallbladder, bladder, spleen, liver, and kidney. The botanical drug’s features are encoded using one-hot encoding for the properties, flavors, and meridian tropisms. As illustrated in Equation 1, where y denotes the botanical drug feature, n represents the total number of properties, flavors, and meridian tropisms, and x refers to a specific property, flavor, or meridian tropism.

$$y_{j}=\left\{\begin{array}{c} 1,j=x\\ 0,j\neq x \end{array}\right.\;j\in \left\{1,2,\ldots n\right\}$$ (1)

A prescription consists of multiple botanical drugs, each possessing distinct properties, flavors, meridian tropisms, and indications. The overall effect of the prescription results from the combined effects of the individual botanical drugs. Consequently, the prescription’s feature is derived by integrating the characteristics of the botanical drugs it contains. This integration involves summing the characteristics of the botanical drugs and then standardizing the result. As shown in Equation 2, m represents the number of botanical drugs contained in the prescription. y denotes the characteristic of each botanical drug. μ represents the mean of the original characteristics of the prescription. σ refers to the s standard deviation of the prescription’s original feature and z represents the standardized prescription feature.

$$z_{i}=\frac{\sum_{i=1}^{m}y_{i}-\mu }{\sigma }$$ (2)

SMILES and protein sequences provide precise representations of the composition and topological structure of molecules. They are extensively utilized and studied in cheminformatics. Extracting molecular features using SMILES and protein sequences is a well-established and efficient approach (Wang et al., 2024). Recently, natural language processing (NLP) has gained increasing prominence in bioinformatics (Pang et al., 2024). Notable NLP models, such as chemical bidirectional encoder representations from transformers (ChemBERT) (Chithrananda et al., 2020), have demonstrated significant success in molecular characterization. The BERT model has also been utilized in research on TCM (Chen et al., 2024). In this study, we extract features from the SMILES of metabolites and target protein sequences using the unsupervised SimCSE model. The SimCSE model generates positive samples through the dropout layer. For a batch of text samples ${\left\{x_{i}\right\}}_{i=1}^{m}$ , each sample is converted into a feature vector using two BERT models, denoted as $h_{i}^{z}=f_{\theta }\left(x_{i},z\right)$ and $h_{i}^{z^{\prime }}=f_{\theta }\left(x_{i},z^{\prime }\right)$ , where z denotes the randomly applied dropout mask and m the number of samples in the batch. The unsupervised SimCSE model employs contrastive learning to fine-tune the BERT model. For our task, SMILES and protein sequences are input into two separate BERT models. Positive samples are generated through the dropout layer. During this process, some features are randomly discarded, and all remaining dissimilar samples are treated as negative samples.

The optimization objective of SimCSE is presented in Equation 3, where τ denotes the temperature hyperparameter. The similarity measure, denoted as sim , is typically calculated using the cosine similarity function, as illustrated in Equation 4.

$$L_{Unsup-SimCSE}=-\log\frac{e^{sim\left(h_{i}^{z_{i}},h_{i}^{z_{i}^{\prime }}\right)/\tau }}{\sum_{j=1}^{N}e^{sim\left(h_{i}^{z_{i}},h_{j}^{z_{j}^{\prime }}\right)/\tau }}$$ (3)

$$sim\left(h_{i},h_{i}^{+}\right)=\frac{h_{i}h_{i}^{+}}{{∥h}_{i}∥\cdot ∥h_{i}^{+}∥}$$ (4)

Additionally, for metabolites with SMILES sequences, Morgan fingerprint features are a key method for characterization (Xu et al., 2023). This technique converts molecules into numerical representations using a specific algorithm, effectively capturing their structural information. As a result, besides extracting features via the SimCSE model, we also obtain Morgan fingerprint features using the RDKit tool to further enhance the botanical drug characteristics.

#### 2.6.2 Data augmentation

Data augmentation is essential for TCM related data, which is typically inherited from historical sources, resulting in a slow growth rate and a relatively small scale (Tian et al., 2024). In this study, prescriptions are used as the predicted samples, with the total number of samples only in the thousands. Additionally, some prescription efficacies exhibit significant imbalance, with a positive-to-negative ratio as high as 1:3.6. To tackle these challenges, we apply data augmentation technique to address both the limited data size and the imbalance in the data distribution.

Data augmentation is a widely used technique in deep learning. In image recognition, data can be enhanced using methods like flipping, rotation, scaling, cropping, color transformation, noise addition, and adversarial generation (Gong et al., 2024). In text classification, common augmentation strategies include synonym replacement, random shuffling, and adversarial text generation (Bayer et al., 2023).

In this part, we employ a GAN-based method for augmenting heterogeneous graph data. GAN consists of two modules: a generator and a discriminator. During training, these metabolites compete with each other, thereby enhancing their performance. The primary goal of GAN is to progressively improve the accuracy of both the generator ( G ) and the discriminator ( D ) ultimately producing synthetic data that closely resembles real data. In GAN, given the input noise z , $G\left(z\right)$ denotes the output generated from the noise. $D\left(x\right)$ indicates the discriminator’s evaluation of real data, while $D\left(G\left(z\right)\right)$ represents the discriminator’s evaluation of the generated data. The discriminator aims to make $D\left(x\right)$ as close to 1 as possible and $D\left(G\left(z\right)\right)$ as close to 0 as possible, whereas the generator strives to make $D\left(G\left(z\right)\right)$ approach. The loss function of GAN is given in Equation 5. x represents real data, and z denotes the input noise. $p_{data}$ refers to the distribution of real data, while $p_{z}\left(z\right)$ represents the distribution of the random noise variable z . $E_{x∼p_{data\left(x\right)}}$ indicates the expectation of obtaining real data x by sampling from the real data distribution $p_{data}$ . $V\left(D,G\right)$ represents the value function. The training process of GAN involves minimizing and maximizing $V\left(D,G\right)$ .

$$\min_{G}\max_{D}V\left(D,G\right)=E_{x∼p_{\text{data}}\left(x\right)}\left[\log D\left(x\right)\right]+E_{z∼p_{z}\left(z\right)}\left[\log1-D\left(G\left(z\right)\right))\right]$$ (5)

In this study, since prescriptions are trained within a heterogeneous graph, it is essential to generate edges for prescription nodes. This is crucial for enabling iterative learning of prescription node features during the subsequent training process of the heterogeneous graph. Therefore, using GAN, we generate three types of data related to prescription efficacy: the labels of prescription nodes, the initial features of prescription nodes, and the adjacency matrix between prescription and botanical drug nodes. To enhance the reliability of GAN-generated data, we prune all prescriptions containing botanical drug pairs explicitly prohibited by the Eighteen Incompatibles and Nineteen Antagonisms incompatibility principles. Then the dataset is subsequently integrated into the original heterogeneous network.

#### 2.6.3 Message passing

In this section, we employ RGAT to perform message passing and integrate node features within a heterogeneous graph. RGAT comprises two key metabolites: an attention mechanism and a relational graph network module. It utilizes multi-head attention to aggregate neighboring nodes and update each node’s features. For a graph G with n nodes, the attention mechanism is represented in Equations 6, 7.

$$h_{{att}_{i}}^{l+1}={\|}_{k=1}^{K}\sum_{j\in N_{i}}\alpha_{ij}^{lk}w_{k}^{l}h_{j}^{l}$$ (6)

$$\alpha_{ij}^{lk}=attention\left(i,j\right)$$ (7)

$\alpha_{ij}^{lk}$ denotes the attention of node i at layer l+1 . The symbol ${\|}_{k=1}^{K}$ represents the concatenation of vectors from $x_{1}$ to $x_{k}$ . $\alpha_{ij}^{lk}$ refers to the normalized attention coefficient calculated by the *k*th attention at layer l . $W_{k}^{l}$ represents the input transformation matrix. $attention\left(i,j\right)$ represents the attention coefficient between the i -th and j -th nodes. $h_{{att}_{i}}^{l+1}$ indicates the feature of node i transmitted to node j at layer l+1 . The calculation formula is provided in Equation 8.

$$attention\left(Q,K,V\right)=softmax\left(\frac{QK^{T}}{\sqrt{d_{k}}}\right)V$$ (8)

Q , K , and V denote the query, key, and value matrices, respectively. Their dimensions are $\left(n,d_{k}\right)$ , $\left(m,d_{k}\right)$ , and $\left(m,d_{v}\right)$ respectively, where n represents the number of queries, m indicates the number of key-value pairs, $d_{k}$ refers to the feature dimension both the keys and queries, and $d_{v}$ represents the feature dimension of values.

Additionally, RGAT uses relational neural networks to regulate the influence of neighboring nodes, each with distinct dependency relationships on the node itself. Initially, different relationships are mapped to separate vectors, followed by the calculation of relational attention, as outlined in Equations 9–11.

$$g_{ij}^{lm}=\sigma \left(relu\left(r_{ij}W_{m1}+b_{m1}\right)W_{m2}+b_{m2}\right)$$ (9)

$$\beta_{ij}^{lm}=\frac{\exp\left(g_{ij}^{lm}\right)}{\sum_{j=1}^{N_{i}}\exp\left(g_{ij}^{lm}\right)}$$ (10)

$$h_{{rel}_{i}}^{l+1}={\|}_{m=1}^{M}\beta_{ij}^{lm}W_{m}^{k}h_{j}^{l}$$ (11)

$r_{ij}$ denotes the relationship embedding between node i and node j . $g_{ij}^{lm}$ refers to the attention weight of the corresponding relationship between node i and node j . $N_{i}$ represents the set of neighbor nodes of node i . $\beta_{ij}^{lm}$ indicates the normalized attention weight. $h_{{rel}_{i}}^{l+1}$ represents the relationship feature transmitted from the *i*th neighboring node to node j at layer l+1 . The node and relationship feature are concatenated to obtain the hybrid node feature. Subsequently, the updated node feature is obtained via the fully connected layer and activation function, as shown in Equations 12, 13.

$$x_{i}^{l+1}=h_{{att}_{i}}^{l+1}\|h_{{rel}_{i}}^{l+1}$$ (12)

$$h_{i}^{l+1}=relu\left(W_{l+1}x_{i}^{l+1}+b_{l+1}\right)$$ (13)

#### 2.6.4 Prescription efficacy prediction

After learning the neighborhood features via RGAT, we extract the feature R for each prescription node. Subsequently, it undergoes standardization and is passed through a linear layer. Finally, the model’s output is obtained through the softmax function, as shown in Equation 14.

$$y^{\prime }=Softmax\left(Liner\left(LayerNorm\left(R\right)\right)\right)$$ (14)

$y^{\prime }$ represents the predicted score for whether a particular prescription has that efficacy or not. The higher of the two values is selected as the prediction result for the prescription efficacy classification, as shown in Equation 15.

$$y=\mathrm{argmax}\left(y^{\prime }\right)$$ (15)

Binary cross-entropy (BCE) is employed as the loss function, as indicated in Equation 16.

$$Loss=-\left(1-y\right)\log\left(1-x\right)-ylog\left(x\right)$$ (16)

## 3 Results

### 3.1 Contrast experiment

At present, few studies have addressed the prediction of TCM prescription efficacy. We have chosen several widely used machine learning and deep learning prediction methods to compare with our model.

- Random Forest (Breiman, 2001)

Random forest is an ensemble learning method that generates predictions by combining the outputs of multiple decision trees. We use the prescription features directly as input to predict the efficacy of prescriptions.

- XGBoost (Chen and Guestrin, 2016)

XGBoost is a machine learning method that employs the gradient boosting framework and has demonstrated strong performance in various regression and classification tasks. We use the same prescription features as for random forest as the input to XGBoost.

- MLP (Taud and Mas, 2018)

MLP, a feedforward neural network, is primarily used to model nonlinear relationships between input and output. It consists of multiple neurons, each connected to others via weights and undergoing nonlinear transformations through an activation function. The output is then passed to the subsequent layer of neurons or the output layer.

- KAN (Liu et al., 2024)

The Kolmogorov-arnold network (KAN) represents an innovative architecture for neural networks. It incorporates learnable activation functions on the weights to enhance the model’s flexibility and expressive capability while preserving its interpretability.

- RGCN (Chen et al., 2019)

The Relational Graph Convolutional Network (RGCN) is a graph neural network designed for processing relational data. It accounts for various types of relationships between nodes and utilizes distinct weight matrices for information propagation, allowing it to more effectively handle complex graph data.

To thoroughly evaluate the model’s performance while minimizing the risk of data leakage during data augmentation, we perform 10-fold cross-validation on the dataset. During the experiment, we maintain an 8:1:1 ratio for the training, validation, and test sets. The code is executed on the Ubuntu 20.04 system, and the programming language used is Python 3.7. We use commonly adopted evaluation metrics in classification tasks, including AUC, area under the precision versus recall curve (AUPR), accuracy, recall, precision, and F1 score, to evaluate the effectiveness of different models. The input features for the remaining several models all employ the initial features of the prescription nodes in the heterogeneous graph. Since the data distribution varies across different efficacy aspects, we perform efficacy prediction on separate datasets. For the balanced dataset, the “regulating blood” efficacy aspect is selected. For the slightly imbalanced dataset, we choose the “regulating qi-flowing” efficacy aspect. For the highly imbalanced dataset, we focus on the “clearing heat” efficacy aspect. Additionally, efficacy prediction is conducted on the severely imbalanced dataset after data augmentation. As shown in Table 3, the numbers of positive and negative samples for the three efficacy aspects are presented. The prediction results of our model, along with those of the comparison models, for various efficacy aspects are presented in Tables 4–7. For each efficacy aspect, our model demonstrates superior performance, achieving optimal results across most evaluation metrics. Specifically, in the four datasets, the AUC and AUPR values are 0.853 and 0.805, 0.796 and 0.659, 0.791 and 0.539, and 0.811 and 0.649, respectively. In the first three datasets, as the degree of data imbalance increases, the model’s performance progressively declines. This indicates that the balance of data significantly impacts the model’s performance in the prescription efficacy prediction task. Additionally, after data augmentation, most models show noticeable improvement in performance. Although the performance does not reach that achieved with balanced samples, the results suggest that data augmentation helps improve the model’s generalization ability, particularly when there is a limited number of efficacy samples and the distribution is imbalanced. However, an analysis of the model’s mispredicted prescriptions reveals its inherent limitations. For instance, in the case of the Phlegm-Resolving and Stasis-Removing Mixture (Huatan Quyu Mixture), the model incorrectly predicted that it possesses blood-regulating efficacy. This error may stem from the model’s tendency to associate the blood-regulating effect primarily with the botanical drug Conioselinum anthriscoides ‘Chuanxiong’ [Apiaceae; Chuanxiong rhizoma]. It indicates that the model fails to capture sufficiently discriminative features from the data and relies excessively on the apparent characteristics of individual botanical drugs. This also underscores the nonlinear fitting relationship between botanical drug combinations and their corresponding efficacy. Moving forward, incorporating more diverse feature dimensions such as the monarch, minister, assistant, and guide roles of botanical drugs and their dosages could help improve both the precision and recall of the model, as well as botanical drug dosage could help improve both prediction accuracy and recall.

**TABLE 4:** Comparing the Performance of TCMRGAT with Other Methods on the balanced data.

Model	Auc	Aupr	Acc	Recall	Precision	F1
Our	0.853 (0.023)	0.805 (0.046)	0.843 (0.023)	0.832 (0.056)	0.821 (0.011)	0.826 (0.013)
Random forest	0.782 (0.012)	0.719 (0.027)	0.782 (0.012)	0.772 (0.032)	0.785 (0.03)	0.778 (0.019)
XGBoost	0.778 (0.009)	0.713 (0.019)	0.777 (0.009)	0.772 (0.033)	0.778 (0.025)	0.774 (0.011)
MLP	0.742 (0.033)	0.671 (0.044)	0.74 (0.035)	0.768 (0.077)	0.726 (0.053)	0.742 (0.037)
KAN	0.535 (0.085)	0.517 (0.072)	0.539 (0.085)	0.49 (0.364)	0.415 (0.238)	0.449 (0.286)
RGCN	0.804 (0.016)	0.738 (0.033)	0.804 (0.015)	0.789 (0.041)	0.807 (0.046)	0.796 (0.014)

**TABLE 5:** Comparing the Performance of TCMRGAT with Other Methods on the slightly unbalanced data.

Model	Auc	Aupr	Acc	Recall	Precision	F1
Our	0.796 (0.03)	0.659 (0.034)	0.775 (0.066)	0.687 (0.114)	0.648 (0.073)	0.667 (0.031)
Random forest	0.681 (0.027)	0.507 (0.049)	0.75 (0.022)	0.464 (0.047)	0.701 (0.071)	0.556 (0.047)
XGBoost	0.688 (0.029)	0.496 (0.043)	0.734 (0.027)	0.544 (0.036)	0.625 (0.058)	0.581 (0.043)
MLP	0.635 (0.039)	0.446 (0.041)	0.691 (0.061)	0.459 (0.162)	0.597 (0.104)	0.489 (0.081)
KAN	0.506 (0.051)	0.347 (0.03)	0.602 (0.051)	0.205 (0.148)	0.272 (0.158)	0.229 (0.147)
RGCN	0.676 (0.03)	0.459 (0.033)	0.675 (0.066)	0.687 (0.113)	0.528 (0.073)	0.585 (0.032)

**TABLE 6:** Comparing the performance of TCMRGAT with other methods on the severely unbalanced data.

Model	Auc	Aupr	Acc	Recall	Precision	F1
Our	0.791 (0.021)	0.539 (0.037)	0.863 (0.005)	0.662 (0.052)	0.703 (0.046)	0.679 (0.027)
Random forest	0.604 (0.038)	0.277 (0.069)	0.884 (0.016)	0.218 (0.077)	0.755 (0.124)	0.333 (0.099)
XGBoost	0.661 (0.029)	0.307 (0.057)	0.880 (0.011)	0.361 (0.06)	0.601 (0.089)	0.448 (0.063)
MLP	0.707 (0.084)	0.427 (0.096)	0.837 (0.041)	0.483 (0.181)	0.661 (0.1)	0.533 (0.148)
KAN	0.521 (0.063)	0.235 (0.042)	0.766 (0.04)	0.099 (0.131)	0.324 (0.299)	0.128 (0.149)
RGCN	0.781 (0.020)	0.537 (0.039)	0.793 (0.005)	0.662 (0.053)	0.654 (0.047)	0.657 (0.027)

**TABLE 7:** Comparing the Performance of TCMRGAT with Other Methods on the Severely Unbalanced Samples Which have been Processed by Data Augmentation.

Model	Auc	Aupr	Acc	Recall	Precision	F1
Our	0.811 (0.011)	0.649 (0.017)	0.855 (0.004)	0.763 (0.061)	0.701 (0.027)	0.731 (0.054)
Random forest	0.701 (0.025)	0.528 (0.026)	0.743 (0.025)	0.558 (0.037)	0.663 (0.031)	0.606 (0.033)
XGBoost	0.692 (0.027)	0.510 (0.022)	0.727 (0.025)	0.572 (0.045)	0.625 (0.026)	0.597 (0.033)
MLP	0.625 (0.081)	0.434 (0.051)	0.66 (0.025)	0.509 (0.377)	0.567 (0.095)	0.533 (0.213)
KAN	0.544 (0.039)	0.374 (0.032)	0.61 (0.035)	0.327 (0.179)	0.408 (0.084)	0.339 (0.142)
RGCN	0.801 (0.023)	0.544 (0.022)	0.853 (0.019)	0.723 (0.041)	0.694 (0.011)	0.708 (0.113)

* The bold values represent the optimal result for each performance metric.

### 3.2 Hyperparameter tuning

In general, model performance is highly influenced by hyperparameter settings (Bischl et al., 2023). In this study, we perform parameter tuning experiments on both balanced prescription data (regulating blood) and unbalanced datasets (clearing heat) using the evaluation metrics mentioned earlier. Our observations indicate that factors such as the length of metabolite and target features, the number of layers and hidden neurons in the graph attention network, and the learning rate ratio between the generator and discriminator in the GAN all significantly affect model performance.

**FIGURE 2:**
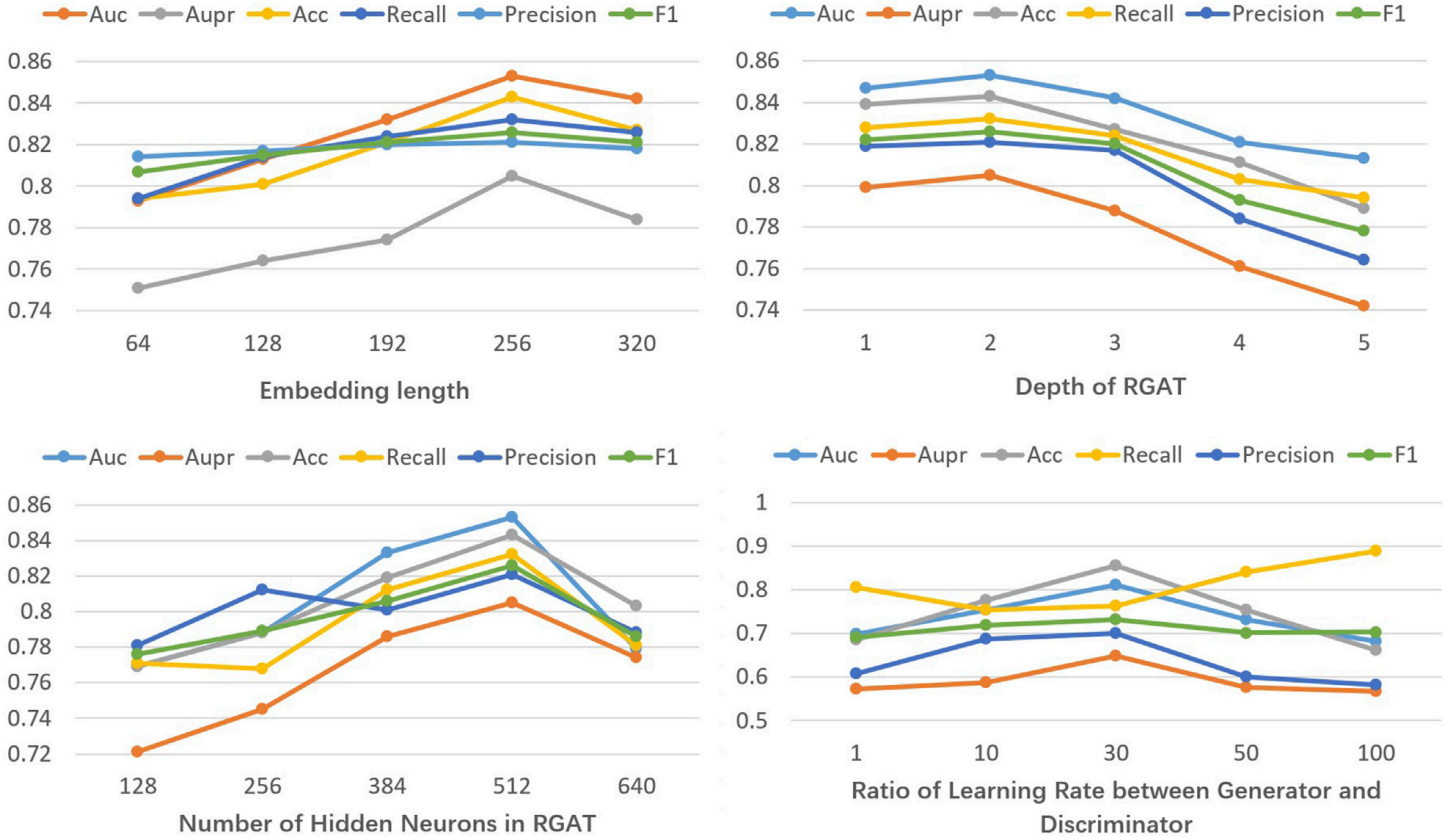
Line Chart of different parameter results.

We define the length range of metabolite and target features as [64, 320], the number of RGAT layers as [1, 5], and the range for hidden neurons as [128, 640]. The step sizes are respectively set to 64, 1, and 128, reflecting values where significant changes in results are observed. Parameter tuning experiments are conducted using the balanced dataset. Figure 2 demonstrates that the model performs optimally on the balanced dataset with a metabolite and target feature length of 256, two RGAT layers, and 512 hidden neurons. Next, with the previously optimized parameters fixed, we perform parameter tuning for GAN on the unbalanced dataset. The learning rate ratio of the generator to the discriminator is set within the range [1, 100], with the discriminator’s learning rate fixed at 1e-5. Figure 2 illustrates that the model performs optimally on the unbalanced dataset when the generator-discriminator ratio is 30. This may be attributed to the fact that at this ratio, the data generated by GAN closely resembles real data.

### 3.3 Ablation experiment

We conduct ablation experiments at each stage, from heterogeneous graph construction to model evaluation, using the existing prescription efficacy dataset. These experiments are designed to evaluate the impact of each module on the model’s overall performance. The experiments are as follows:

1. TCMRGAT-R: Data related to metabolite is excluded during the heterogeneous graph construction, meaning the relationships between metabolite-target and botanical drug-metabolite are omitted.
2. TCMRGAT-E: The features of metabolites and targets are extracted using the BERT model instead of the SimCSE model.
3. TCMRGAT-H: Prescription features are obtained by concatenating botanical drug features.
4. TCMRGAT-S: For the unbalanced dataset, the SMOTE method is applied to augment prescription samples. However, since SMOTE-enhanced samples do not generate an adjacency matrix, Random Forest is employed for prediction.

The experimental results shown in Table 8, 9 reveal a noticeable decline in the predictive performance of various model variants. Specifically, for the balanced data, TCMRGAT-R exhibits the most significant performance degradation, underscoring the importance of the metabolite-target and botanical drug-metabolite relationships in prescription efficacy prediction. Furthermore, the performance of TCMRGAT-E and TCMRGAT-H also decreases slightly, suggesting that our feature extraction approach more accurately captures node representations. In the case of the unbalanced dataset, the performance of TCMRGAT-S is also considerably reduced. This suggests that the data augmentation method we use offers improved generalization in prescription efficacy prediction for unbalanced data, compared to traditional data augmentation techniques.

**TABLE 8:** Ablation experiments under the balanced dataset.

Model variant	Auc	Aupr	Acc	Recall	Precision	F1
TCMRGAT	0.853 (0.023)	0.805 (0.046)	0.843 (0.023)	0.832 (0.056)	0.821 (0.011)	0.826 (0.013)
TCMRGAT-R	0.779 (0.141)	0.761 (0.047)	0.754 (0.042)	0.745 (0.044)	0.764 (0.041)	0.752 (0.022)
TCMRGAT-E	0.832 (0.094)	0.794 (0.067)	0.821 (0.033)	0.812 (0.047)	0.811 (0.085)	0.811 (0.022)
TCMRGAT-H	0.844 (0.032)	0.789 (0.024)	0.831 (0.042)	0.822 (0.097)	0.815 (0.014)	0.818 (0.022)

**TABLE 9:** Ablation experiments under the unbalanced dataset.

Model variant	Auc	Aupr	Acc	Recall	Precision	F1
TCMRGAT	0.811 (0.011)	0.649 (0.017)	0.855 (0.004)	0.763 (0.061)	0.701 (0.027)	0.731 (0.054)
TCMRGAT-S	0.766 (0.034)	0.469 (0.042)	0.833 (0.035)	0.650 (0.047)	0.604 (0.052)	0.625 (0.044)

^*^ The bold values represent the optimal result for each performance metric.

### 3.4 Case study

To better validate our model’s performance in real-world scenarios, we selected two classical prescriptions, Longgui Decoction and Zhenxuan Decoction, as a dedicated test set. The complete compositions of these prescriptions are detailed in Table 10. The predicted prescription efficacy encompasses those that are filtered out during data cleaning on account of the exceedingly small number of positive samples. Regarding these efficacies with a minuscule number of positive samples, we conduct data augmentation prior to making predictions. Recently, large language models have been increasingly applied in TCM research (Zhou et al., 2024). In this part, we utilize the generative pretrained transformer-4‌ (GPT-4) model to predict prescription efficacy. The prompt words for GPT-4 are shown in Table 11, and the prediction results are presented in Table 12. As shown in the results, our model demonstrates high accuracy in predicting actual prescription efficacies. Moreover, its prediction accuracy surpasses that of the GPT-4 model, further confirming the practical application potential of our model.

**TABLE 10:** Composition of the test set prescriptions.

Prescription	Constituent drug
Longgui decoction	• Pheretima aspergillum [Megascolecidae; Pheretima] • Angelica sinensis (Oliv.) Diels [Apiaceae; Angelicae sinensis radix] • Hirudo nipponica [Hirudinidae; Hirudo] • Conioselinum anthriscoides ‘Chuanxiong’ [Apiaceae; Chuanxiong rhizoma] • Panax notoginseng (Burkill) F.H.Chen [Araliaceae; Notoginseng radix et rhizoma] • Astragalus mongholicus Bunge [Fabaceae; Astragali mongholici radix] • Gastrodia elata Blume [Orchidaceae; Gastrodiae rhizoma • Lycium barbarum L. [Solanaceae; Lycii fructus] • Chrysanthemum indicum L. [Asteraceae; Chrysanthemi flos] • Alisma plantago-aquatica subsp. orientale (Sam.) Sam. [Alismataceae; Alismatis rhizoma] • Wolfiporia cocos [Polyporaceae; Poria]
Zhenxuan decoction	• Conioselinum anthriscoides ‘Chuanxiong’ [Apiaceae; Chuanxiong rhizoma] • Paeonia lactiflora Pall. [Paeoniaceae; Paeoniae radix] • Angelica sinensis (Oliv.) Diels [Apiaceae; Angelicae sinensis radix] • Rehmannia glutinosa (Gaertn.) Libosch. Ex DC. [Orobanchaceae; Rehmanniae radix] • Neolitsea cassia (L.) Kosterm. [Lauraceae; Cinnamomi cortex] • Wolfiporia cocos [Polyporaceae; Poria] • Atractylodes macrocephala Koidz. [Asteraceae; Atractylodis macrocephalae rhizoma] • Glycyrrhiza glabra L. [Fabaceae; Glycyrrhizae radix et rhizoma] • Fossilia Ossis Mastodi [Fossil; Os draconis] • Concha Ostreae [Animal; Concha ostreae cruda]

**TABLE 11:** GPT-4 Prescription prediction prompt for botanical drug prescriptions.

Prescription	Prompt
LongGui decoction	Based on Traditional Chinese Medicine theory and modern research, analyze the core therapeutic actions of the following botanical drug formula - Prescription Name: Longgui Decoction - Composition: Pheretima 15g, angelicae sinensis radix 15g, hirudo 6g, chuanxiong rhizoma 12g, notoginseng radix 3g, astragali radix 30g, gastrodiae rhizoma 12g, lycii fructus 15g, chrysanthemi flos 15g, alismatis rhizoma 30g, poria 30 g
Zhenxuan decoction	Based on Traditional Chinese Medicine theory and modern research, analyze the core therapeutic actions of the following botanical drug formula - Prescription Name: Zhenxuan Decoction - Composition: Chuanxiong rhizome 10–16g, paeoniae radix alba 10–16g, angelicae sinensis radix 10–12g, rehmanniae radix praeparata 10–12g, cinnamomi ramulus 10–12g, poria alba 12–18g, atractylodis macrocephalae rhizoma 10g, glycyrrhizae radix 10g, fossilia ossis mastodi 30–60g, concha ostreae cruda 30–60 g

**TABLE 12:** Comparison of TCMRGAT output and GPT-4 model in specific cases.

Type	LongGui decoction	Zhenxuan decoction
Real	1 Expelling phlegm 2 Eliminating dampness 3 Regulating qi-flowing 4 Invigoration 5 Regulating blood	1 Eliminating dampness 2 Expelling phlegm 3 Dispelling wind 4 Regulating blood 5 Resolving food
Our	✓ Expelling phlegm ✓ Regulating qi-flowing ✓ Invigoration ✓ Regulating blood ● Dispelling wind	✓ Eliminating dampness ✓ Expelling phlegm ✓ Dispelling wind ✓ Regulating blood ● Invigoration
GPT4	✓ Regulating blood ✓ Invigoration ✓ Eliminating dampness ● Dispelling wind ● Improving eyesight ● Tranquillization	✓ Eliminating dampness ✓ Expelling phlegm ✓ Dispelling wind ● Tranquillization

*The prescription efficacy name marked in green indicates that the model predicted correctly.

### 3.5 Analysis on attention weight

**FIGURE 3:**
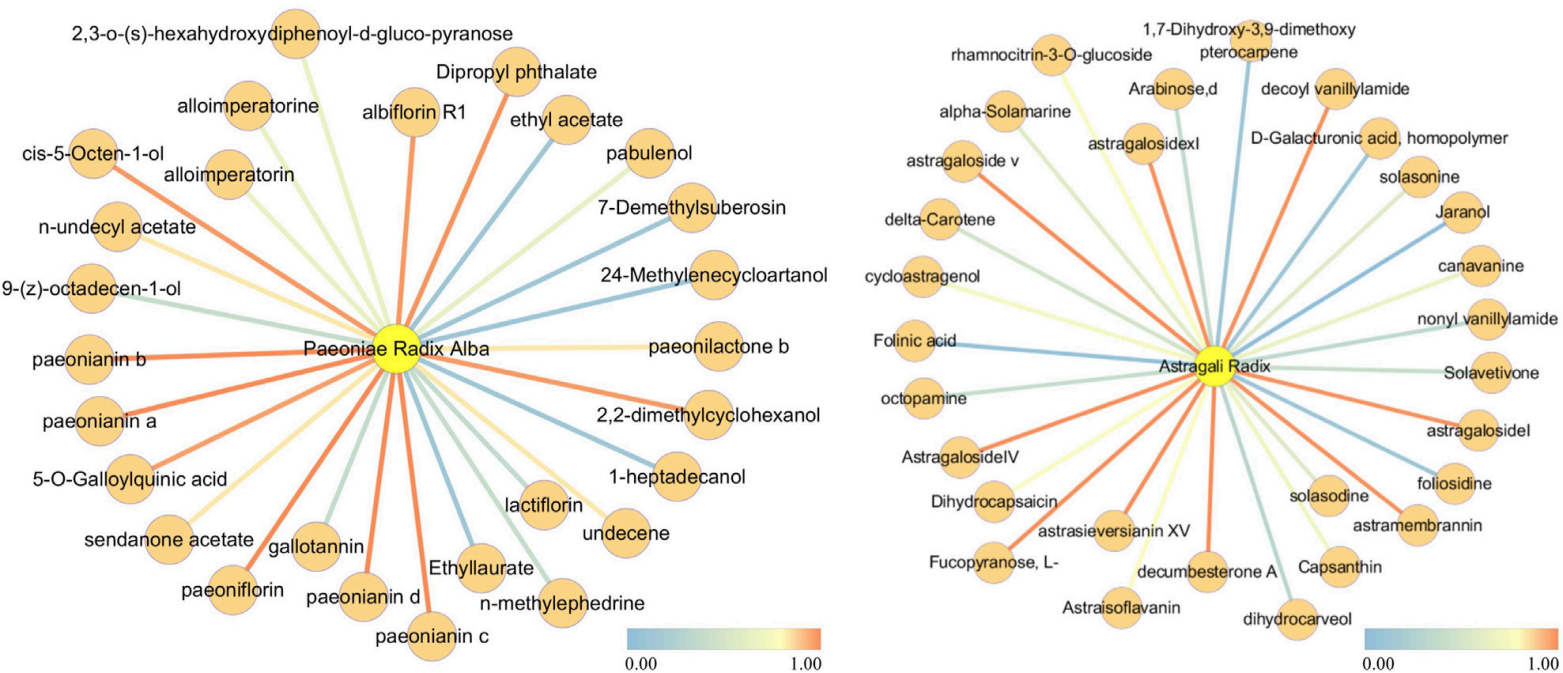
Botanical drug-Metabolite Relationship Network of Astragali Radix and Paeoniae Radix Alba.

In TCM, each botanical drug consists of multiple metabolites. Among these, those responsible for the therapeutic effects are referred to as active metabolites. We analyze the attention weights related to botanical drugs and their metabolites within the heterogeneous attention network to explain the principle of TCM treatment. For this analysis, we choose Astragali Radix and Paeoniae Radix Alba from Buyang Huanwu Decoction and Zhengan Xifeng Decoction. The attention coefficients range from 0 to 1, with increments of 0.1. The node relationships with the top five attention coefficients at each step are extracted, and the resulting attention weights are used as edge weights, as illustrated in Figure 3. It can be observed that the weights between certain nodes are considerably higher following training. For example, the attention weight between Astragali Radix and Astragaloside IV is 0.6987. Astragaloside IV is recognized as one of the active metabolites of Astragali Radix. may exert therapeutic effects in ischemic stroke by downregulating AQP4, which in turn inhibits the upregulation of MMP-9 and suppresses the water flux across brain microvascular endothelium to astrocytes (Li et al., 2013). Similarly, among the metabolites related to Paeoniae Radix Alba, the attention weight of paeoniflorin is also notably higher after training, with an attention coefficient of 0.705. While the precise mechanism of paeoniflorin in stroke prevention and treatment remains unclear, it is well established that paeoniflorin possesses neuroprotective properties and contributes to the treatment of ischemic stroke (Wang et al., 2022). In conclusion, we believe that among the relationships between botanical drug metabolites after training, those metabolite nodes showing a significant increase in weight may be linked to the mechanism of the corresponding botanical drug medicine in stroke treatment.

The left graph illustrates the botanical drug-metabolite relationship network for Astragali Radix, while the right graph illustrates the network for Paeoniae Radix Alba. Edges with a redder hue signify denote relationships with higher attention weights, while those with a bluer hue indicate represent relationships with lower attention weights.

## 4 Discussion and conclusion

In this study, we propose an innovative model for predicting prescription efficacy. The model performs data augmentation using GAN and conducts feature learning by constructing a heterogeneous graph. Specifically, for datasets with imbalanced positive and negative samples, GAN is applied to increase the sample size, thereby alleviating the imbalance issue. A heterogeneous network is then built, incorporating various relationships relevant to prescriptions, such as prescription-botanical drug, botanical drug-metabolite, and metabolite-target. Features are extracted from metabolite and target sequences using the SimCSE model and RDKit tool, respectively. Botanical drugs are encoded based on their properties, flavors, and meridian tropisms, while prescriptions are represented by integrating the features of botanical drugs. Finally, neighborhood learning is performed through a heterogeneous attention network to obtain the feature representation for each node, and a fully connected layer is employed to predict the specific efficacy of the prescriptions.

The experimental data used in this study primarily originates from ancient and modern TCM classics and the HERB database. Comparative experiments have demonstrated the superior performance of the TCMRGAT model in predicting prescription efficacy. Additionally, ablation experiments show that, compared to directly expanding prescription samples using other data augmentation methods, the GAN-based data augmentation approach, which enhances prescription samples and generates their relationships within heterogeneous graphs, achieves better predictive performance. This finding provides valuable insights for future data augmentation efforts in the field of small and imbalanced sample sizes in TCM. Additionally, we conduct case studies on specific prescriptions and compared the results with the GPT-4 model to further validate our model’s performance in predicting prescription efficacy. Finally, by analyzing the variations in attention weights before and after training, we identify that the metabolite nodes with increased attention may be linked to the therapeutic mechanisms of the related botanical drugs in stroke treatment. This finding can provide valuable insights for future research on the treatment mechanisms of prescriptions.

While the TCMRGAT model has shown promising results in predicting prescription efficacy, it has certain limitations. For example, data quality remains an area requiring improvement. Traditional Chinese Medicine data span extensive periods, and data standards are often ambiguous. For instance, the dosages of metabolites within some botanical drug formulae are imprecise, and measurement units lack standardization. During the construction of the heterogeneous network, the dosage variations of distinct botanical drugs across different formulae were not considered. Botanical drug dosage, to a certain extent, reflects the monarch, minister, assistant, guide of botanical drugs within a formula. The absence of this critical attribute prevents models from learning relevant features, consequently leading to degraded model performance. Moreover, although GAN-generated data can partially mitigate the effects of data imbalance, it remains uncontrollable. Specifically, it is not possible to control the positive-to-negative sample ratio or restrict the number of relationships between generated samples in heterogeneous graphs. This can compromise the quality of the generated data and, as a result, hinder the model’s training process. Notably, we do not predict all prescription efficacies. We exclude those with highly imbalanced samples. In the future, we will place greater emphasis on the quality of collected data. All existing data will undergo multi-faceted verification to ensure authenticity and reliability. For data that prove difficult to validate definitively, we plan to incorporate techniques such as graph completion to enhance model robustness, thereby mitigating the impact of data limitations on model performance. Furthermore, we will also focus on data augmentation techniques to improve the quality of generated data in scenarios with extreme sample imbalance, and subsequently predict the remaining efficacies. Building upon the prescription efficacy prediction method, we will further explore a clinical recommendation paradigm for botanical drug combinations. Specifically, we will focus on predicting the efficacy of various botanical drug combinations in relation to patient symptoms, with the goal of recommending potential yet underexplored botanical drug formulations. This approach will also facilitate the iterative refinement of our efficacy prediction method based on clinical performance. We plan to incorporate additional TCM-specific mechanisms, such as the Eight Principles and Six Meridians, to enrich node feature dimensions. These enhanced features will strengthen our capability to elucidate the underlying mechanisms of Traditional Chinese Medicine through graph neural network technology. Finally, we will also pursue methodological improvements. Given that Traditional Chinese Medicine data inherently follows a multimodal distribution, we aim to design a multimodal framework incorporating an adaptive attention mechanism. This framework is intended to enhance model interpretability, and while predicting prescription efficacy, it will simultaneously co-learn to predict the targets and potential side effects of key botanical drug pairs. This approach will allow for a deeper analysis of the mechanistic actions of Traditional Chinese Medicine.

In summary, our study demonstrates that the approach of enhancing data via GAN and employing heterogeneous networks to predict the efficacy of prescriptions holds significant potential. The TCMRGAT model has exhibited outstanding performance in this domain and is anticipated to become an effective tool for future research on TCM prescriptions. However, the model also has several limitations. First, the insufficient quality and standardization of data such as missing or inconsistent dosage information for botanical drugs in prescriptions limit the model’s ability to deeply explore key compatibility principles like the “monarch, minister, assistant, and guide” roles. Second, although GAN has been introduced for data augmentation, the controllability of its generation process still requires improvement. Furthermore, the current model does not cover all types of prescription efficacy, particularly exhibiting limited predictive capability for categories with extreme sample imbalance. These factors collectively constrain further enhancement of the model’s performance and generalization ability. In the future, we will focus on addressing these shortcomings to further advance the contribution of TCMRGAT to research in Traditional Chinese Medicine.

## Data Availability

The original contributions presented in the study are included in the article/supplementary material, further inquiries can be directed to the corresponding author.
